# 4-Chloro-*N*-(3,5-dimethyl­phen­yl)benzene­sulfonamide

**DOI:** 10.1107/S1600536811028819

**Published:** 2011-07-23

**Authors:** K. Shakuntala, Sabine Foro, B. Thimme Gowda

**Affiliations:** a Department of Chemistry, Mangalore University, Mangalagangotri-574 199, Mangalore, India; b Institute of Materials Science, Darmstadt University of Technology, Petersenstrasse 23, D-64287, Darmstadt, Germany

## Abstract

The asymmetric unit of the title compound, C_14_H_14_ClNO_2_S, contains two independent mol­ecules, which are twisted at the S atoms with C—SO_2_—NH—C torsion angles of −69.4 (7)° and 66.0 (8)°. The sulfonyl and the anilino benzene rings are tilted relative to each other by 49.0 (4) and 61.7 (3)° in the two mol­ecules. In the crystal, the mol­ecules are linked into chains by N—H⋯O hydrogen bonds.

## Related literature

For hydrogen-bonding modes of sulfonamides, see: Adsmond & Grant (2001 ▶). For our studies of the effect of substituents on the structures of *N*-(ar­yl)-amides, see: Gowda *et al.* (2006 ▶), on *N*-(ar­yl)aryl­sulfonamides, see: Nirmala *et al.* (2009 ▶); Shakuntala *et al.* (2011**a* ▶,b* ▶) and on *N*-(ar­yl)methane­sulfonamides, see: Gowda *et al.* (2007 ▶).
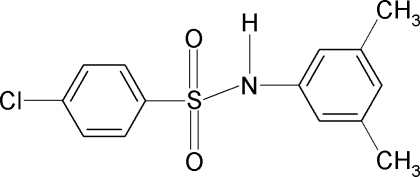


## Experimental

### 

#### Crystal data

C_14_H_14_ClNO_2_S
*M* *_r_* = 295.77
Orthorhombic, 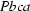

*a* = 21.990 (2) Å
*b* = 10.0470 (8) Å
*c* = 26.408 (2) Å
*V* = 5834.4 (8) Å^3^
*Z* = 16
Mo *K*α radiation
μ = 0.40 mm^−1^
*T* = 293 K
0.40 × 0.20 × 0.06 mm

#### Data collection

Oxford Diffraction Xcalibur diffractometer with Sapphire CCD detector
Absorption correction: multi-scan (*CrysAlis RED*; Oxford Diffraction, 2009 ▶) *T*_min_ = 0.856, *T*_max_ = 0.976
21403 measured reflections
5320 independent reflections
2694 reflections with *I* > 2σ(*I*)
*R*_int_ = 0.106

#### Refinement

*R*[*F* ^2^ > 2σ(*F* ^2^)] = 0.128
*wR*(*F* ^2^) = 0.345
*S* = 1.07
5320 reflections
347 parameters
1 restraint
H-atom parameters constrained
Δρ_max_ = 1.06 e Å^−3^
Δρ_min_ = −0.41 e Å^−3^

### 

Data collection: *CrysAlis CCD* (Oxford Diffraction, 2009 ▶); cell refinement: *CrysAlis RED* (Oxford Diffraction, 2009 ▶); data reduction: *CrysAlis RED*; program(s) used to solve structure: *SHELXS97* (Sheldrick, 2008 ▶); program(s) used to refine structure: *SHELXL97* (Sheldrick, 2008 ▶); molecular graphics: *PLATON* (Spek, 2009 ▶); software used to prepare material for publication: *SHELXL97*.

## Supplementary Material

Crystal structure: contains datablock(s) I, global. DOI: 10.1107/S1600536811028819/ds2123sup1.cif

Structure factors: contains datablock(s) I. DOI: 10.1107/S1600536811028819/ds2123Isup2.hkl

Supplementary material file. DOI: 10.1107/S1600536811028819/ds2123Isup3.cml

Additional supplementary materials:  crystallographic information; 3D view; checkCIF report

## Figures and Tables

**Table 1:** Hydrogen-bond geometry (Å, °)

*D*—H⋯*A*	*D*—H	H⋯*A*	*D*⋯*A*	*D*—H⋯*A*
N1—H1*A*⋯O2^i^	0.86	2.49	3.001 (9)	119
N2—H2*A*⋯O1^ii^	0.86	2.39	3.006 (9)	130

Symmetry codes: (i) 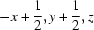; (ii) 
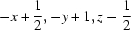
.

**Fig. 1.**
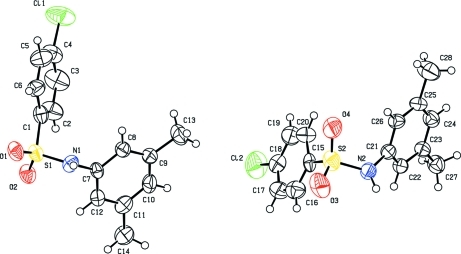
Molecular structure of (I), showing the atom labelling scheme and displacement ellipsoids are drawn at the 50% probability level.

**Fig. 2.**
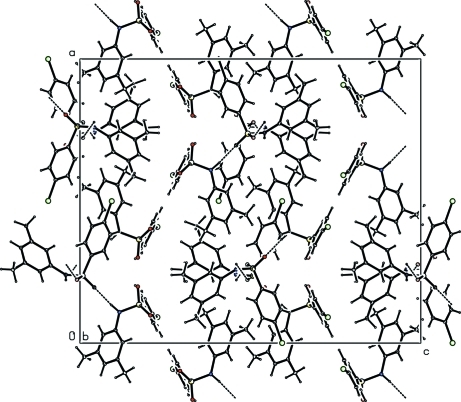
Molecular packing of (I) with hydrogen bonding shown as dashed lines.

## supplementary crystallographic information

### Comment

The sulfonamide moieties are the constituents of many biologically important compounds. The hydrogen bonding preferences of sulfonamides have been investigated (Adsmond & Grant, 2001). As a part of our work on the substituent effects on the structures and other aspects of this class of compounds (Gowda *et al.*, 2006, 2007; Nirmala *et al.*, 2009; Shakuntala *et al.*, 2011**a*,b*), in the present work, the crystal structure of 4-chloro-*N*-(3,5-dimethylphenyl)- benzenesulfonamide (I) has been determined (Fig.1). The asymmetric unit of the structure contains two independent molecules. The molecules are twisted at the S atoms with the C—SO_2_—NH—C torsion angles of -69.5 (7)° and 66.1 (8)° in the two molecules, compared to the values of 65.3 (2)° and 54.6 (2)° in the two independent molecules of 4-chloro-*N*-(2,5-dimethylphenyl)-benzenesulfonamide (II) (Shakuntala *et al.*, 2011*b*), and -53.8 (3)° and -63.4 (3)° in the two molecules of 4-chloro-*N*-(phenyl)-benzenesulfonamide (III) (Shakuntala *et al.*, 2011*a*) and 67.9 (2)° in *N*-(3,5-dimethylphenyl)benzenesulfonamide (IV)(Nirmala *et al.*, 2009).

The sulfonyl and the anilino benzene rings in the two independent molecules of (I) are tilted relative to each other by 49.0 (4)° (molecule 1) and 61.7 (3)° (molecule 2), compared to the values of 59.3 (1)° (molecule 1) and 45.8 (1)° (molecule 2) in (II), -53.8 (3)° and -63.4 (3)° in the two independent molecules of (III), and 54.6 (1)° in (IV)

In the title compound the molecules are linked into chains by N—H···O(S) hydrogen bonding (Table 1 and Fig.2).

### Experimental

The solution of chlorobenzene (10 ml) in chloroform (40 ml) was treated dropwise with chlorosulfonic acid (25 ml) at 0 ° C. After the initial evolution of hydrogen chloride subsided, the reaction mixture was brought to room temperature and poured into crushed ice in a beaker. The chloroform layer was separated, washed with cold water and allowed to evaporate slowly. The residual 4-chlorobenzenesulfonylchloride was treated with 3,5-dimethylaniline in the stoichiometric ratio and boiled for ten minutes. The reaction mixture was then cooled to room temperature and added to ice cold water (100 ml). The resultant 4-chloro-*N*-(3,5-dimethylphenyl)-benzenesulfonamide was filtered under suction and washed thoroughly with cold water. It was then recrystallized to constant melting point from aqueous ethanol. The compound was characterized by recording its infrared and NMR spectra.

Prism like colourless single crystals used in X-ray diffraction studies were grown in ethanolic solution by slow evaporation at room temperature.

### Refinement

The H atoms were positioned with idealized geometry using a riding model with the aromatic C—H = 0.93 Å, methyl C—H = 0.96 Å, N—H = 0.86 Å and were refined with isotropic displacement parameters (set to 1.2 times of the *U*_eq_ of the parent atom).

The residual electron-density features are located in the region of H1A and S1. The highest peak is 1.31 Å from H1A and the deepest hole is 1.09 Å from S1. To improve considerably values of R1, wR2, and GOOF these bad four reflections (4 3 10 2 2 7 2 2 5 2 3 4) were omitted from the refinement.

The crystals available for X-ray studies were of rather poor quality and weak scatterers at high theta value resulting in relatively high *R* values.

### Crystal data

**Table:**

C_14_H_14_ClNO_2_S	*F*(000) = 2464
*M**_r_* = 295.77	*D*_x_ = 1.347 Mg m^−^^3^
Orthorhombic, *P**b**c**a*	Mo *K*α radiation, λ = 0.71073 Å
Hall symbol: -P 2ac 2ab	Cell parameters from 2329 reflections
*a* = 21.990 (2) Å	θ = 2.5–27.8°
*b* = 10.0470 (8) Å	µ = 0.40 mm^−^^1^
*c* = 26.408 (2) Å	*T* = 293 K
*V* = 5834.4 (8) Å^3^	Prism, colourless
*Z* = 16	0.40 × 0.20 × 0.06 mm

### Data collection

**Table:**

Oxford Diffraction Xcalibur diffractometer with Sapphire CCD detector	5320 independent reflections
Radiation source: fine-focus sealed tube	2694 reflections with *I* > 2σ(*I*)
graphite	*R*_int_ = 0.106
Rotation method data acquisition using ω scans.	θ_max_ = 25.4°, θ_min_ = 2.7°
Absorption correction: multi-scan (*CrysAlis RED*; Oxford Diffraction, 2009)	*h* = −26→26
*T*_min_ = 0.856, *T*_max_ = 0.976	*k* = −9→12
21403 measured reflections	*l* = −25→31

### Refinement

**Table:**

Refinement on *F*^2^	Primary atom site location: structure-invariant direct methods
Least-squares matrix: full	Secondary atom site location: difference Fourier map
*R*[*F*^2^ > 2σ(*F*^2^)] = 0.128	Hydrogen site location: inferred from neighbouring sites
*wR*(*F*^2^) = 0.345	H-atom parameters constrained
*S* = 1.07	*w* = 1/[σ^2^(*F*_o_^2^) + (0.1148*P*)^2^ + 41.4199*P*] where *P* = (*F*_o_^2^ + 2*F*_c_^2^)/3
5320 reflections	(Δ/σ)_max_ = 0.003
347 parameters	Δρ_max_ = 1.06 e Å^−^^3^
1 restraint	Δρ_min_ = −0.41 e Å^−^^3^

### Special details

**Table:**

Geometry. All e.s.d.'s (except the e.s.d. in the dihedral angle between two l.s. planes) are estimated using the full covariance matrix. The cell e.s.d.'s are taken into account individually in the estimation of e.s.d.'s in distances, angles and torsion angles; correlations between e.s.d.'s in cell parameters are only used when they are defined by crystal symmetry. An approximate (isotropic) treatment of cell e.s.d.'s is used for estimating e.s.d.'s involving l.s. planes.
Refinement. Refinement of *F*^2^ against ALL reflections. The weighted *R*-factor *wR* and goodness of fit *S* are based on *F*^2^, conventional *R*-factors *R* are based on *F*, with *F* set to zero for negative *F*^2^. The threshold expression of *F*^2^ > σ(*F*^2^) is used only for calculating *R*-factors(gt) *etc*. and is not relevant to the choice of reflections for refinement. *R*-factors based on *F*^2^ are statistically about twice as large as those based on *F*, and *R*- factors based on ALL data will be even larger.

### Fractional atomic coordinates and isotropic or equivalent isotropic displacement parameters (Å^2^)

**Table:**

	*x*	*y*	*z*	*U*_iso_*/*U*_eq_	
Cl1	0.00034 (14)	0.0421 (4)	0.59314 (17)	0.1103 (14)	
S1	0.26317 (10)	0.0246 (2)	0.50757 (9)	0.0454 (6)	
O1	0.3036 (3)	0.0914 (6)	0.5418 (2)	0.0544 (17)	
O2	0.2769 (3)	−0.1061 (6)	0.4911 (3)	0.0547 (17)	
N1	0.2594 (3)	0.1192 (7)	0.4583 (3)	0.0456 (18)	
H1A	0.2778	0.1947	0.4587	0.055*	
C1	0.1902 (4)	0.0237 (9)	0.5340 (3)	0.047 (2)	
C2	0.1472 (5)	−0.0646 (11)	0.5157 (5)	0.070 (3)	
H2	0.1570	−0.1267	0.4910	0.084*	
C3	0.0889 (5)	−0.0574 (12)	0.5355 (5)	0.082 (4)	
H3	0.0593	−0.1163	0.5242	0.098*	
C4	0.0746 (5)	0.0348 (11)	0.5713 (5)	0.065 (3)	
C5	0.1174 (5)	0.1204 (11)	0.5888 (4)	0.069 (3)	
H5	0.1075	0.1818	0.6138	0.083*	
C6	0.1749 (4)	0.1166 (10)	0.5699 (4)	0.056 (3)	
H6	0.2039	0.1770	0.5814	0.068*	
C7	0.2264 (3)	0.0807 (8)	0.4140 (3)	0.037 (2)	
C8	0.1732 (4)	0.1481 (9)	0.4019 (3)	0.044 (2)	
H8	0.1583	0.2126	0.4239	0.053*	
C9	0.1421 (4)	0.1212 (10)	0.3577 (4)	0.049 (2)	
C10	0.1645 (4)	0.0205 (10)	0.3270 (4)	0.054 (2)	
H10	0.1435	−0.0003	0.2974	0.065*	
C11	0.2166 (5)	−0.0504 (10)	0.3383 (4)	0.057 (3)	
C12	0.2471 (4)	−0.0189 (9)	0.3823 (3)	0.049 (2)	
H12	0.2822	−0.0655	0.3907	0.058*	
C13	0.0873 (4)	0.2025 (12)	0.3421 (4)	0.074 (3)	
H13A	0.0989	0.2941	0.3383	0.088*	
H13B	0.0563	0.1954	0.3676	0.088*	
H13C	0.0717	0.1697	0.3105	0.088*	
C14	0.2416 (5)	−0.1558 (11)	0.3035 (4)	0.070 (3)	
H14A	0.2404	−0.1242	0.2693	0.084*	
H14B	0.2174	−0.2350	0.3064	0.084*	
H14C	0.2828	−0.1754	0.3128	0.084*	
Cl2	0.09120 (19)	0.4163 (3)	0.22721 (16)	0.1050 (13)	
S2	0.13516 (12)	1.0127 (3)	0.17349 (10)	0.0600 (8)	
O3	0.1989 (3)	1.0367 (8)	0.1698 (3)	0.085 (2)	
O4	0.0976 (4)	1.0924 (8)	0.2066 (3)	0.081 (2)	
N2	0.1092 (3)	1.0317 (8)	0.1159 (3)	0.0504 (19)	
H2A	0.1342	1.0514	0.0920	0.061*	
C15	0.1242 (4)	0.8416 (10)	0.1887 (3)	0.048 (2)	
C16	0.1665 (5)	0.7509 (12)	0.1776 (4)	0.065 (3)	
H16	0.2030	0.7759	0.1625	0.078*	
C17	0.1549 (5)	0.6177 (11)	0.1893 (4)	0.069 (3)	
H17	0.1832	0.5524	0.1809	0.083*	
C18	0.1036 (5)	0.5848 (11)	0.2123 (4)	0.062 (3)	
C19	0.0606 (5)	0.6750 (12)	0.2248 (4)	0.071 (3)	
H19	0.0243	0.6488	0.2398	0.085*	
C20	0.0720 (4)	0.8036 (11)	0.2150 (4)	0.058 (3)	
H20	0.0447	0.8683	0.2258	0.070*	
C21	0.0456 (4)	1.0162 (9)	0.1041 (3)	0.042 (2)	
C22	0.0300 (4)	0.9218 (9)	0.0686 (3)	0.044 (2)	
H22	0.0596	0.8654	0.0556	0.053*	
C23	−0.0305 (4)	0.9104 (9)	0.0518 (3)	0.046 (2)	
C24	−0.0726 (4)	0.9957 (9)	0.0725 (4)	0.052 (2)	
H24	−0.1127	0.9909	0.0615	0.063*	
C25	−0.0572 (4)	1.0907 (9)	0.1101 (4)	0.050 (2)	
C26	0.0030 (4)	1.0975 (9)	0.1251 (3)	0.044 (2)	
H26	0.0144	1.1584	0.1498	0.053*	
C27	−0.0480 (4)	0.8106 (10)	0.0130 (4)	0.057 (3)	
H27A	−0.0597	0.7292	0.0293	0.068*	
H27B	−0.0141	0.7940	−0.0090	0.068*	
H27C	−0.0815	0.8440	−0.0066	0.068*	
C28	−0.1049 (5)	1.1804 (13)	0.1316 (5)	0.086 (4)	
H28A	−0.1370	1.1277	0.1459	0.104*	
H28B	−0.1210	1.2359	0.1052	0.104*	
H28C	−0.0872	1.2352	0.1575	0.104*	

### Atomic displacement parameters (Å^2^)

**Table:**

	*U*^11^	*U*^22^	*U*^33^	*U*^12^	*U*^13^	*U*^23^
Cl1	0.0679 (19)	0.107 (3)	0.156 (4)	−0.008 (2)	0.046 (2)	0.006 (3)
S1	0.0432 (12)	0.0429 (13)	0.0501 (13)	0.0036 (10)	−0.0105 (10)	−0.0032 (11)
O1	0.046 (4)	0.060 (4)	0.057 (4)	0.001 (3)	−0.022 (3)	−0.009 (3)
O2	0.065 (4)	0.039 (4)	0.061 (4)	0.008 (3)	−0.010 (3)	−0.001 (3)
N1	0.047 (4)	0.053 (5)	0.037 (4)	−0.011 (3)	−0.007 (3)	−0.006 (4)
C1	0.049 (5)	0.041 (5)	0.051 (5)	−0.003 (4)	−0.013 (4)	0.000 (5)
C2	0.053 (6)	0.066 (7)	0.091 (8)	−0.006 (5)	−0.001 (6)	−0.022 (6)
C3	0.058 (7)	0.069 (8)	0.119 (11)	−0.021 (6)	−0.001 (7)	−0.015 (8)
C4	0.059 (6)	0.046 (6)	0.091 (8)	0.005 (5)	0.013 (6)	0.011 (6)
C5	0.076 (8)	0.064 (7)	0.067 (7)	−0.007 (6)	0.013 (6)	−0.013 (6)
C6	0.054 (6)	0.067 (7)	0.048 (6)	−0.004 (5)	0.002 (5)	−0.005 (5)
C7	0.031 (4)	0.036 (5)	0.044 (5)	−0.004 (4)	−0.008 (4)	0.005 (4)
C8	0.043 (5)	0.046 (5)	0.043 (5)	0.001 (4)	−0.004 (4)	−0.005 (4)
C9	0.038 (5)	0.063 (6)	0.046 (5)	0.002 (4)	0.000 (4)	0.006 (5)
C10	0.053 (6)	0.065 (7)	0.044 (5)	−0.002 (5)	−0.014 (4)	0.004 (5)
C11	0.066 (6)	0.061 (6)	0.045 (6)	−0.003 (5)	0.000 (5)	−0.006 (5)
C12	0.049 (5)	0.053 (6)	0.044 (5)	0.008 (5)	−0.006 (4)	0.004 (5)
C13	0.049 (6)	0.094 (9)	0.079 (8)	0.009 (6)	−0.012 (5)	0.016 (7)
C14	0.080 (7)	0.075 (8)	0.054 (6)	0.009 (6)	−0.010 (6)	−0.021 (6)
Cl2	0.124 (3)	0.075 (2)	0.116 (3)	−0.007 (2)	−0.004 (2)	0.035 (2)
S2	0.0554 (15)	0.0633 (17)	0.0614 (16)	−0.0026 (13)	−0.0178 (13)	−0.0092 (14)
O3	0.048 (4)	0.096 (6)	0.111 (6)	−0.020 (4)	−0.028 (4)	0.009 (5)
O4	0.103 (6)	0.087 (6)	0.052 (4)	0.022 (5)	−0.020 (4)	−0.028 (4)
N2	0.038 (4)	0.068 (5)	0.046 (4)	−0.007 (4)	−0.002 (3)	0.007 (4)
C15	0.049 (5)	0.053 (6)	0.041 (5)	0.004 (5)	−0.019 (4)	0.002 (5)
C16	0.061 (6)	0.072 (8)	0.063 (7)	−0.004 (6)	−0.001 (5)	−0.012 (6)
C17	0.075 (8)	0.063 (7)	0.070 (8)	0.021 (6)	0.010 (6)	−0.004 (6)
C18	0.069 (7)	0.069 (7)	0.049 (6)	0.004 (6)	0.001 (5)	0.008 (5)
C19	0.058 (6)	0.079 (6)	0.074 (8)	−0.003 (6)	0.003 (6)	0.017 (7)
C20	0.051 (6)	0.066 (5)	0.059 (6)	0.005 (5)	0.010 (5)	−0.012 (5)
C21	0.041 (5)	0.042 (5)	0.044 (5)	−0.002 (4)	0.004 (4)	0.007 (4)
C22	0.044 (5)	0.046 (5)	0.043 (5)	0.005 (4)	0.005 (4)	0.004 (4)
C23	0.037 (5)	0.052 (6)	0.048 (5)	0.000 (4)	0.001 (4)	0.002 (5)
C24	0.042 (5)	0.057 (6)	0.057 (6)	0.000 (4)	−0.002 (4)	0.004 (5)
C25	0.042 (5)	0.058 (6)	0.050 (6)	0.010 (4)	0.009 (4)	−0.005 (5)
C26	0.045 (5)	0.046 (6)	0.042 (5)	0.000 (4)	0.003 (4)	−0.009 (4)
C27	0.054 (6)	0.058 (6)	0.058 (6)	0.002 (5)	−0.008 (5)	−0.005 (5)
C28	0.065 (7)	0.093 (9)	0.102 (10)	0.019 (7)	−0.003 (7)	−0.021 (8)

### Geometric parameters (Å, °)

**Table:**

Cl1—C4	1.732 (10)	Cl2—C18	1.760 (11)
S1—O2	1.416 (6)	S2—O3	1.425 (7)
S1—O1	1.434 (6)	S2—O4	1.445 (8)
S1—N1	1.613 (7)	S2—N2	1.636 (7)
S1—C1	1.750 (9)	S2—C15	1.782 (10)
N1—C7	1.429 (10)	N2—C21	1.442 (10)
N1—H1A	0.8600	N2—H2A	0.8600
C1—C6	1.374 (13)	C15—C16	1.335 (13)
C1—C2	1.383 (13)	C15—C20	1.394 (13)
C2—C3	1.384 (14)	C16—C17	1.396 (15)
C2—H2	0.9300	C16—H16	0.9300
C3—C4	1.361 (16)	C17—C18	1.323 (15)
C3—H3	0.9300	C17—H17	0.9300
C4—C5	1.357 (15)	C18—C19	1.351 (15)
C5—C6	1.360 (13)	C19—C20	1.342 (14)
C5—H5	0.9300	C19—H19	0.9300
C6—H6	0.9300	C20—H20	0.9300
C7—C12	1.382 (12)	C21—C26	1.361 (11)
C7—C8	1.390 (11)	C21—C22	1.378 (12)
C8—C9	1.380 (12)	C22—C23	1.406 (12)
C8—H8	0.9300	C22—H22	0.9300
C9—C10	1.387 (13)	C23—C24	1.376 (12)
C9—C13	1.513 (12)	C23—C27	1.485 (12)
C10—C11	1.382 (13)	C24—C25	1.417 (13)
C10—H10	0.9300	C24—H24	0.9300
C11—C12	1.377 (12)	C25—C26	1.383 (12)
C11—C14	1.505 (13)	C25—C28	1.495 (13)
C12—H12	0.9300	C26—H26	0.9300
C13—H13A	0.9600	C27—H27A	0.9600
C13—H13B	0.9600	C27—H27B	0.9600
C13—H13C	0.9600	C27—H27C	0.9600
C14—H14A	0.9600	C28—H28A	0.9600
C14—H14B	0.9600	C28—H28B	0.9600
C14—H14C	0.9600	C28—H28C	0.9600
			
O2—S1—O1	119.7 (4)	O3—S2—O4	120.6 (5)
O2—S1—N1	108.0 (4)	O3—S2—N2	105.0 (5)
O1—S1—N1	105.4 (4)	O4—S2—N2	107.4 (4)
O2—S1—C1	108.2 (4)	O3—S2—C15	108.2 (5)
O1—S1—C1	108.6 (4)	O4—S2—C15	108.7 (5)
N1—S1—C1	106.1 (4)	N2—S2—C15	106.0 (4)
C7—N1—S1	121.8 (6)	C21—N2—S2	121.8 (6)
C7—N1—H1A	119.1	C21—N2—H2A	119.1
S1—N1—H1A	119.1	S2—N2—H2A	119.1
C6—C1—C2	120.6 (9)	C16—C15—C20	119.8 (10)
C6—C1—S1	119.7 (7)	C16—C15—S2	120.9 (8)
C2—C1—S1	119.5 (8)	C20—C15—S2	119.2 (8)
C3—C2—C1	117.9 (10)	C15—C16—C17	118.6 (10)
C3—C2—H2	121.1	C15—C16—H16	120.7
C1—C2—H2	121.1	C17—C16—H16	120.7
C4—C3—C2	120.8 (10)	C18—C17—C16	119.7 (10)
C4—C3—H3	119.6	C18—C17—H17	120.1
C2—C3—H3	119.6	C16—C17—H17	120.1
C5—C4—C3	120.4 (10)	C17—C18—C19	122.8 (11)
C5—C4—Cl1	121.0 (9)	C17—C18—Cl2	118.4 (9)
C3—C4—Cl1	118.6 (9)	C19—C18—Cl2	118.7 (9)
C4—C5—C6	120.3 (11)	C20—C19—C18	117.8 (11)
C4—C5—H5	119.9	C20—C19—H19	121.1
C6—C5—H5	119.9	C18—C19—H19	121.1
C5—C6—C1	119.9 (10)	C19—C20—C15	120.9 (10)
C5—C6—H6	120.0	C19—C20—H20	119.5
C1—C6—H6	120.0	C15—C20—H20	119.5
C12—C7—C8	119.3 (8)	C26—C21—C22	121.3 (8)
C12—C7—N1	121.7 (7)	C26—C21—N2	121.0 (8)
C8—C7—N1	119.0 (8)	C22—C21—N2	117.6 (8)
C9—C8—C7	121.2 (8)	C21—C22—C23	120.4 (8)
C9—C8—H8	119.4	C21—C22—H22	119.8
C7—C8—H8	119.4	C23—C22—H22	119.8
C8—C9—C10	117.4 (8)	C24—C23—C22	117.3 (9)
C8—C9—C13	121.3 (9)	C24—C23—C27	121.5 (8)
C10—C9—C13	121.2 (9)	C22—C23—C27	121.2 (8)
C11—C10—C9	122.9 (9)	C23—C24—C25	122.6 (8)
C11—C10—H10	118.5	C23—C24—H24	118.7
C9—C10—H10	118.5	C25—C24—H24	118.7
C12—C11—C10	117.9 (9)	C26—C25—C24	117.5 (8)
C12—C11—C14	119.9 (9)	C26—C25—C28	122.2 (9)
C10—C11—C14	122.2 (9)	C24—C25—C28	120.3 (9)
C11—C12—C7	121.2 (9)	C21—C26—C25	120.8 (8)
C11—C12—H12	119.4	C21—C26—H26	119.6
C7—C12—H12	119.4	C25—C26—H26	119.6
C9—C13—H13A	109.5	C23—C27—H27A	109.5
C9—C13—H13B	109.5	C23—C27—H27B	109.5
H13A—C13—H13B	109.5	H27A—C27—H27B	109.5
C9—C13—H13C	109.5	C23—C27—H27C	109.5
H13A—C13—H13C	109.5	H27A—C27—H27C	109.5
H13B—C13—H13C	109.5	H27B—C27—H27C	109.5
C11—C14—H14A	109.5	C25—C28—H28A	109.5
C11—C14—H14B	109.5	C25—C28—H28B	109.5
H14A—C14—H14B	109.5	H28A—C28—H28B	109.5
C11—C14—H14C	109.5	C25—C28—H28C	109.5
H14A—C14—H14C	109.5	H28A—C28—H28C	109.5
H14B—C14—H14C	109.5	H28B—C28—H28C	109.5
			
O2—S1—N1—C7	46.5 (7)	O3—S2—N2—C21	−179.5 (7)
O1—S1—N1—C7	175.5 (6)	O4—S2—N2—C21	−50.1 (8)
C1—S1—N1—C7	−69.4 (7)	C15—S2—N2—C21	66.0 (8)
O2—S1—C1—C6	153.8 (7)	O3—S2—C15—C16	−23.8 (9)
O1—S1—C1—C6	22.4 (9)	O4—S2—C15—C16	−156.4 (8)
N1—S1—C1—C6	−90.5 (8)	N2—S2—C15—C16	88.4 (8)
O2—S1—C1—C2	−30.9 (9)	O3—S2—C15—C20	152.8 (8)
O1—S1—C1—C2	−162.3 (8)	O4—S2—C15—C20	20.2 (9)
N1—S1—C1—C2	84.8 (9)	N2—S2—C15—C20	−95.0 (8)
C6—C1—C2—C3	−1.1 (16)	C20—C15—C16—C17	5.0 (15)
S1—C1—C2—C3	−176.4 (9)	S2—C15—C16—C17	−178.4 (8)
C1—C2—C3—C4	0.8 (19)	C15—C16—C17—C18	−2.1 (17)
C2—C3—C4—C5	−0.9 (19)	C16—C17—C18—C19	0.9 (18)
C2—C3—C4—Cl1	178.0 (10)	C16—C17—C18—Cl2	−179.0 (9)
C3—C4—C5—C6	1.3 (18)	C17—C18—C19—C20	−2.5 (18)
Cl1—C4—C5—C6	−177.5 (9)	Cl2—C18—C19—C20	177.3 (9)
C4—C5—C6—C1	−1.7 (17)	C18—C19—C20—C15	5.4 (17)
C2—C1—C6—C5	1.6 (15)	C16—C15—C20—C19	−6.8 (15)
S1—C1—C6—C5	176.9 (8)	S2—C15—C20—C19	176.5 (9)
S1—N1—C7—C12	−71.4 (10)	S2—N2—C21—C26	62.2 (11)
S1—N1—C7—C8	110.6 (8)	S2—N2—C21—C22	−121.8 (8)
C12—C7—C8—C9	−2.7 (13)	C26—C21—C22—C23	2.1 (13)
N1—C7—C8—C9	175.3 (8)	N2—C21—C22—C23	−173.9 (8)
C7—C8—C9—C10	2.7 (13)	C21—C22—C23—C24	−0.5 (13)
C7—C8—C9—C13	−174.6 (9)	C21—C22—C23—C27	179.1 (8)
C8—C9—C10—C11	−1.5 (14)	C22—C23—C24—C25	−1.2 (14)
C13—C9—C10—C11	175.9 (10)	C27—C23—C24—C25	179.2 (9)
C9—C10—C11—C12	0.2 (15)	C23—C24—C25—C26	1.2 (14)
C9—C10—C11—C14	−177.6 (9)	C23—C24—C25—C28	−179.6 (10)
C10—C11—C12—C7	−0.2 (15)	C22—C21—C26—C25	−2.1 (14)
C14—C11—C12—C7	177.7 (9)	N2—C21—C26—C25	173.8 (8)
C8—C7—C12—C11	1.4 (13)	C24—C25—C26—C21	0.4 (14)
N1—C7—C12—C11	−176.6 (9)	C28—C25—C26—C21	−178.7 (10)

### Hydrogen-bond geometry (Å, °)

**Table:**

*D*—H···*A*	*D*—H	H···*A*	*D*···*A*	*D*—H···*A*
N1—H1A···O2^i^	0.86	2.49	3.001 (9)	119.
N2—H2A···O1^ii^	0.86	2.39	3.006 (9)	130.

Symmetry codes: (i) −*x*+1/2, *y*+1/2, *z*; (ii) −*x*+1/2, −*y*+1, *z*−1/2.
